# Development of a GFP-labeled *Pasteurella multocida* strain for *in vivo* organ infection tracing using a stable RCR-based genetic manipulation plasmid

**DOI:** 10.1128/aem.00787-26

**Published:** 2026-08-12

**Authors:** Chen Yuan, Wenqing Wang, Zhihui Wu, Haoshuai Song, Hongjie Fan, Zhe Ma

**Affiliations:** 1 Ministry of Agriculture Key Laboratory of Animal Bacteriology, the International Joint Laboratory of Animal Health and Food Safety, and College of Veterinary Medicine, Nanjing Agricultural University 70578, Nanjing, Jiangsu, China; 2 Jiangsu Co-innovation Center for Prevention and Control of Important Animal Infectious Diseases and Zoonoses, Yangzhou, China; Universidad de los Andes, Bogotá, Colombia

**Keywords:** swine-derived *Pasteurella multocida*, markerless genetic manipulation, RCR replicon, *in vivo *pathogen tracking

## Abstract

Advances in microscopy have enabled the widespread application of *in vivo* pathogen tracking approaches to bacterial pathogenesis. *Pasteurella multocida* (*P. multocida*) is a major zoonotic pathogen causing severe respiratory diseases in livestock. However, genetic manipulation of clinical swine-derived isolates remains hindered by the lack of stable genetic tools. As a result, no genetically engineered *in vivo* tracking strain limits research in *P. multocida* infection dynamics. In this study, we evaluated diverse replication mechanisms and identified that rolling circle replication (RCR)-type replicons exhibit superior transformation robustness in swine isolates. Leveraging the temperature-sensitive pSET4s backbone and an optimized *mpheS* counter-selection marker, we developed a high-efficiency, markerless genetic manipulation system. The system exhibited high copy numbers and significantly enhanced transformation yields across diverse clinical swine isolates. To validate gene knockout efficiency, we performed markerless deletions of the capsule biosynthesis genes *hyaD* and *hyaE*. Phenotypic characterization revealed that hyaluronic acid (HA) deficiency led to complete attenuation of virulence in a murine model. Furthermore, we engineered a 15-Pm::GFP reporter strain via site-specific gene knock-in without detectable effects on bacterial growth or virulence. This strain enables direct visualization of bacterial colonization in the lung and liver by fluorescence imaging and allows real-time, *in situ* monitoring of pathogen and Kupffer cell interactions. Collectively, our results established an effective genetic toolkit for swine-derived *P. multocida* and developed fluorescently labeled strains for *in vivo* tracking. This plasmid provides a useful tool for investigating pathogenic mechanisms and *in vivo* infection dynamics of *P. multocida*.

**IMPORTANCE:**

*P. multocida* is a critical threat to global animal health, while the difficulty of genetic modification in swine isolates has interrupted the link of the genomic and pathogenic phenotypes. This study provides a highly efficient, RCR-based markerless manipulation system optimized with cross-serogroup applicability for swine isolates. Validated across 15 clinical swine isolates, this plasmid offers a robust tool for genetic analysis and the rational design of next-generation vaccines. By defining the essential roles of *hyaD* and *hyaE*, we demonstrate that the hyaluronic acid capsule is required for virulence and pulmonary colonization. Moreover, the development of a biologically fluorescent reporter strain enables high-resolution, *in situ* tracking of infection dynamics, providing a powerful tool for real-time visualization during *P. multocida* infection.

## INTRODUCTION

*Pasteurella multocida* (*P. multocida*) is a zoonotic pathogen responsible for a broad spectrum of respiratory diseases in human, livestock, and poultry (1, 2). Based on the capsular and lipopolysaccharide (LPS) antigens, *P. multocida* is classified into five capsular serogroups (A, B, D, E, and F) and 16 LPS serovars (3, 4). Among these, serogroup A exhibits the highest prevalence across diverse hosts and serves as the primary cause of swine pneumonia, avian cholera, and rabbit coryza, inflicting substantial economic losses on the global livestock industry (5). The capsule of serogroup A is composed of hyaluronic acid (HA), structurally identical to that of *Streptococcus pyogenes*, which facilitates immune evasion and tissue colonization (6–8). Although high-throughput genomic sequencing has elucidated the complex genetic landscape of swine-derived *P. multocida*, functional genomic studies for clinical swine isolates have lagged (9–11).

A universal gene-editing toolkit is required for assessing virulence mechanisms. While several plasmids have been adapted for avian-derived *P. multocida* genetics, significant variability in transformation efficiency and plasmid copy number exists among clinical swine isolates (12–14). In this study, we utilized the broad-host-range vector pSET4s (15), derived from *Streptococcus* and featuring a temperature-sensitive replication origin (pWV01), as a backbone to develop a stable and efficient plasmid for further integration in swine-derived serogroup A strains. Recently, a markerless manipulation system carrying the mutated phenylalanyl-tRNA synthetase ɑ-subunit gene (*pheSm*) was established and efficiently used in avian-derived *P. multocida* isolates (16). This system relies on the lethal incorporation of the phenylalanine analog *p-Cl-Phe* into cellular proteins, offering superior screening efficiency and broader applicability across diverse strains (17). Building upon these advancements, we integrated the *mPheS* cassette into the pSET4s backbone to develop the pSET4s-mPheS plasmid. Compared to traditional low-copy suicide plasmids, this system offers superior cloning efficiency in swine isolates, establishing a foundation for markerless genomic editing.

Fluorescent labeling has become an indispensable tool for investigating bacterial pathogenesis and host-pathogen interactions (18). For instance, *Vibrio harveyi* strains tagged with GFP or RFP have been developed for co-infection experiments to track real-time colonization and competitive dynamics (19), which are characterized by high brightness and stability at the single-cell level and within opaque tissues (20). Although fluorescent labeling via constitutive expression plasmids has been reported (21), issues such as plasmid instability and insufficient signal intensity often preclude effective *in situ* observation within complex tissues. These limitations often preclude long-term *in situ* observation within complex host environments. Consequently, the development of a stable, high-intensity fluorescent tracking strain for swine-derived *P. multocida* is significant for real-time monitoring of pathogen distribution and host-pathogen interactions within host microenvironments.

In this study, we established a pSET4s-mPheS-based markerless gene-editing system with broad applicability across clinical swine-derived *P. multocida* isolates. Using this system, we successfully generated deletion mutants of the capsule biosynthesis genes *hyaD* and *hyaE*. Furthermore, we engineered a high-brightness 15-Pm::GFP reporter strain by employing site-specific genomic integration. Collectively, our work not only establishes a high-efficiency genetic manipulation plasmid for porcine *P. multocida* but also provides an *in vivo* imaging tool for the real-time, *in situ* monitoring of host-pathogen interactions.

## RESULTS

### RCR-type replicons show compatibility in swine-derived *P. multocida* serogroup A 15-Pm

To establish a suitable genetic manipulation plasmid for *Pasteurella multocida* (*P. multocida*) serogroup A isolated from pigs, we systematically evaluated the compatibility of replicons in different plasmids, including *Enterobacteriaceae*-derived *θ*-type and rolling circle replication (RCR)-type. Initial antibiotic sensitivity assays showed that the swine-derived *P. multocida* serogroup A 15-Pm strain was highly sensitive to the antibiotics corresponding to the selectable resistance markers used in the plasmids for genetic manipulation, including spectinomycin, chloramphenicol, ampicillin, and kanamycin, enabling reliable transformant selection.

**Fig 1:**
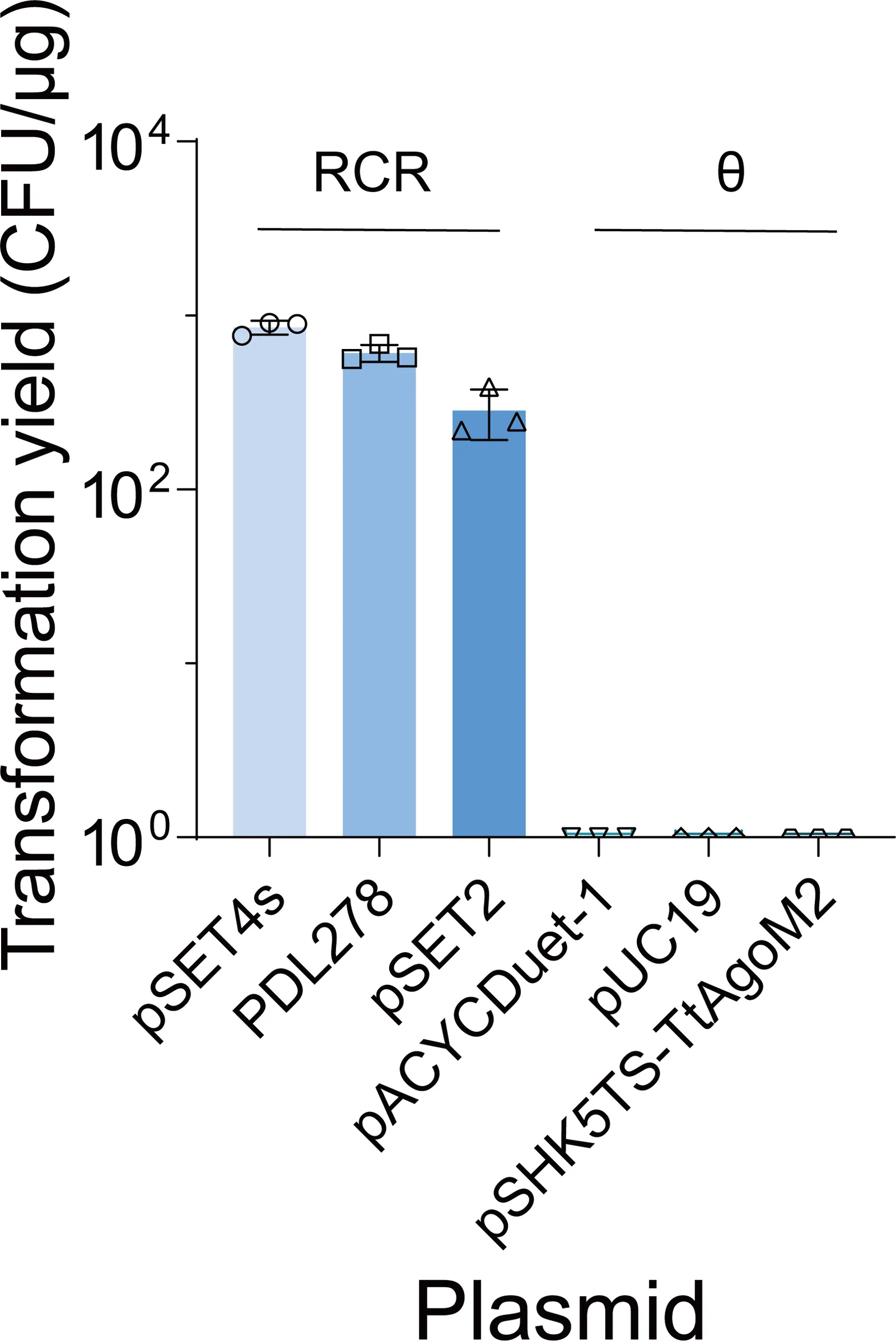
Systematic evaluation of plasmid replicon compatibility in a tested clinical swine-derived serogroup A *P. multocida*. Comparison of transformation efficiencies between RCR-type and *Enterobacteriaceae*-derived *θ*-type replication in the 15-Pm isolate. Data are presented as the mean ± SD from three independent experiments.

Plasmids were electroporated into 15-Pm, and replicon compatibility was determined by growth of transformants on selective antibiotic plates. The pACYCDuet-1 (p15A origin), pUC19, and pSHK5TS-TtAgoM2-HF (ColE1-like origin) plasmids that harbor *Enterobacteriaceae*-derived *θ*-type replication failed to yield any viable transformants across multiple trials (Fig. 1A). Previous studies successfully utilized shuttle vectors like pAL99 or pPBA1100 in avian-derived *P. multocida* (13, 14), which specifically rely on specialized endogenous *P. multocida* plasmid replicons. In contrast, shuttle vectors equipped with RCR-type replicons exhibited remarkable compatibility. The pWV01-based pSET4s and the Inc18-family replicon-based plasmids (pDL278 and pSET2) were successfully transformed into 15-Pm with sufficient transformant count and supported robust growth of transformants on the corresponding antibiotic-selective plates (Fig. 1A). The compatibility of RCR-based plasmids with the 15-Pm strain may be attributed to its host-independent replication mechanism. Plasmid-encoded functional proteins that drive rolling-circle replication work efficiently within serogroup A 15-Pm, thereby facilitating stable replication. Notably, pSET4s carries superior performance due to its temperature-sensitive (Ts) *repA*, allowing stable maintenance at 28°C and rapid loss at 37°C, making it an ideal backbone for markerless genetic manipulation in 15-Pm.

### Establishment and optimization of an mpheS-based markerless mutagenesis system

**Fig 2:**
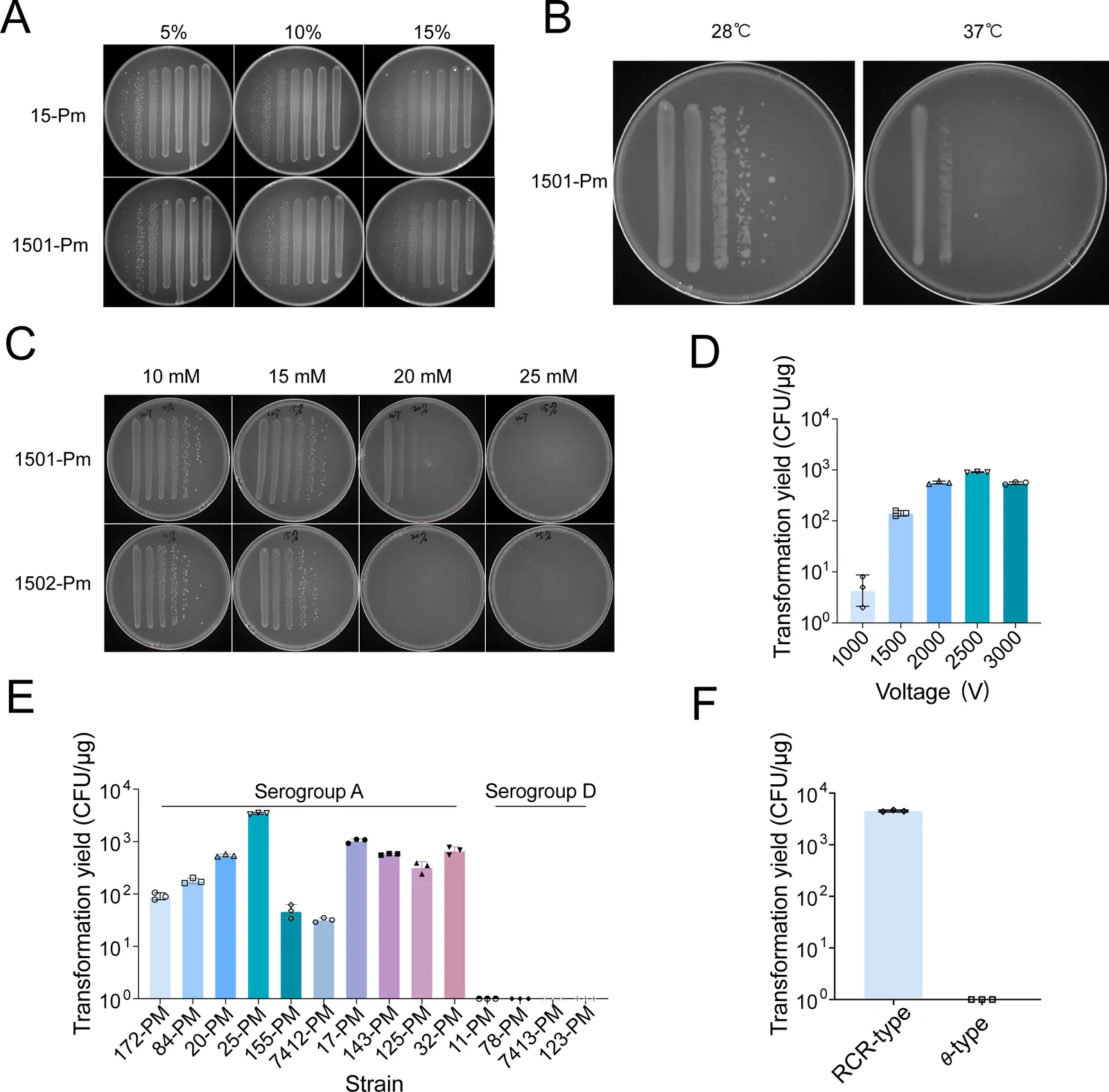
Development and functional validation of a temperature-sensitive markerless mutagenesis system based on the *mPheS* counter-selection marker. (**A**) Assessment of *sacB*-mediated sucrose sensitivity. Growth of WT 15-Pm and 1501-Pm (carrying pSET4s-sacB) was compared on TSA plates supplemented with increasing concentrations of sucrose (5%–15%, wt/vol). (**B**) Functional validation of *mPheS* as a counterselection marker in 15-Pm. Growth inhibition of WT 15-Pm and 1502-Pm (carrying pSET4s-mPheS) was assessed on TSA plates supplemented with p-Cl-Phe at concentrations from 10 to 25 mM. (**C**) Characterization of the temperature-sensitive properties of the pSET4s backbone in 15-Pm. CFU counts of 1501-Pm were determined at permissive (28°C) and non-permissive (37°C) temperatures to evaluate plasmid stability and clearance. (**D**) Optimization of electroporation parameters. Electrotransformation efficiency of pSET4s-mPheS was evaluated under diverse voltage conditions from 1,000 to 3,000 V. (**E**) Evaluation of RCR-type plasmid transformation yields under common protocols used in serogroup A. Transformation yields were determined across diverse serogroup A (*n* = 10) and serogroup D (*n* = 4) clinical swine-derived isolates of *P. multocida*. (**F**) Effects of capsule digestion on serogroup D transformation yields. Following heparinase III digestion to clear the extracellular heparosan capsule of strain 11-Pm, transformation yields were compared between the RCR-type plasmid pSET4s and the *Enterobacteriaceae*-derived *θ*-type plasmid pET28a. Data in panels D to F are expressed as the mean ± SD from three independent experiments.

To develop a markerless mutagenesis plasmid for swine-derived *P. multocida* serogroup A, we evaluated the efficiency of two common counter-selection markers, *sacB* and *mpheS*, integrated into the temperature-sensitive pSET4s backbone (Supplemented Fig. S1A). The correct assembly and structural integrity of these two engineered vectors were successfully confirmed via PCR-based verification (Fig. S1B and C). The pSET4s-sacB plasmid was electroporated into 15-Pm to generate the 1501-Pm strain. This strain was not inhibited by sucrose concentrations up to 15% (wt/vol) on TSA plates (Fig. 2A). In contrast, a ~100-fold reduction in CFU was observed at 37°C compared to 28°C on TSA plates with 100 µg/mL spectinomycin (Fig. 2B), indicating that although the *sacB* system is ineffective for counterselection in *P. multocida* 15-Pm, the temperature-sensitive (Ts) component of the pSET4s backbone is highly efficient in this strain.

The *mPheS* system exhibited efficient counterselection activity. The 1502-Pm strain carrying the pSET4s-mPheS plasmid displayed dose-dependent growth inhibition in response to increasing concentrations of p-chlorophenylalanine (p-Cl-Phe), indicating its suitability as a counterselectable marker in the 15-Pm strain (Fig. 2C). Electroporation conditions were further optimized, with 2,500 V yielding maximal electrotransformation efficiency (Fig. 2D).

Finally, we assessed the general applicability of the pSET4s-mPheS plasmid in 14 swine-derived *P. multocida* strains, which were collected from different regions of China between 2021 and 2024. All 10 serogroup A isolates harboring the pSET4s-mPheS plasmid were able to grow on TSA plates with 100 µg/mL spectinomycin, achieving transformation yields ranging from 10^1^ and 10^4^ CFU/µg DNA (Fig. 2E). Initially, the competent cell preparation protocol used in serogroup A yielded zero transformants for any serogroup D isolates (Fig. 2E). However, by incorporating a targeted capsule-digestion step using heparinase III to remove the extracellular heparosan in serogroup D strain Pm-11, we successfully obtained robust, selectable transformants harboring the pSET4s-mPheS plasmid. In contrast, parallel *Enterobacteriaceae*-derived *θ*-type vectors, such as pET28a (which shares the same origin of replication as pSHK5TS-TtAgoM2-HF), remained completely restricted (Fig. 2F). Collectively, these results suggest that the capsule is an important component for external DNA entry in *P. multocida* and identify that pSET4s-mPheS is a high-performance, broadly applicable toolkit for the genetic manipulation of swine-derived *P. multocida*.

### Construction and characterization of hyaD and hyaE deletion mutants in *P. multocida*

**Fig 3:**
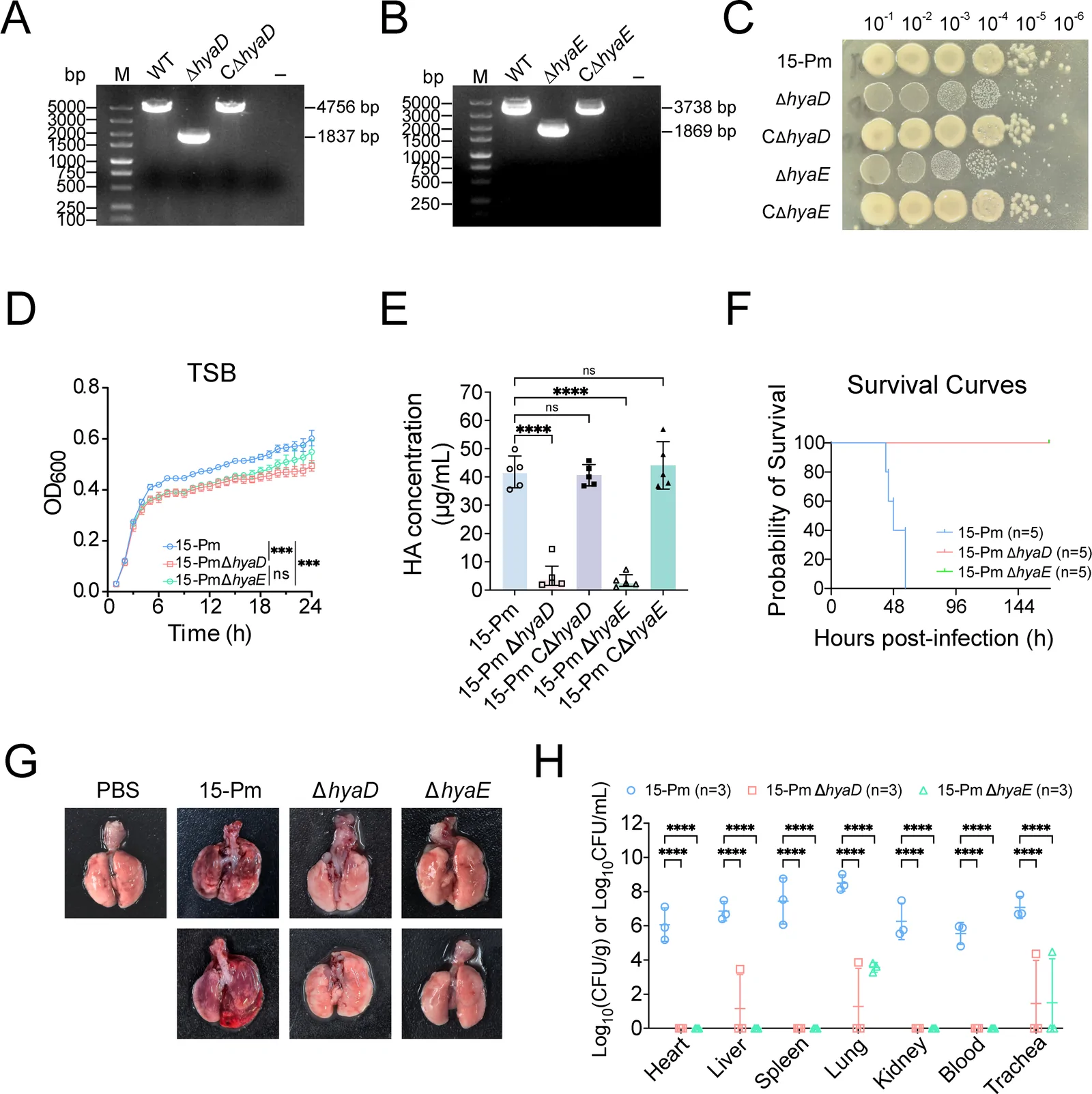
Impact of HA capsule deficiency on the physiological characteristics and systemic virulence of *P. multocida*. (**A**) PCR-based verification of the markerless ∆*hyaD* deletion mutant (1,837 bp) and its corresponding *in situ* complemented strain C∆*hyaD* (4,756 bp). Lane M: 5,000-bp DNA marker; Lane −: negative control. (**B**) PCR-based verification of the markerless ∆*hyaE* deletion mutant (1,869 bp) and its corresponding *in situ* complemented strain C∆*hyaE* (3,738 bp). Lane M: DL 5,000-bp DNA marker; Lane −: negative control. (**C**) Colony morphology of WT 15-Pm and the HA-deficient mutants. 10-fold serial dilutions on TSA plates present the colony morphology transformed from highly mucoid (WT, CΔ*hyaD,* and CΔ*hyaE*) to rough, non-mucoid (Δ*hyaD* and Δ*hyaE*) appearance following capsule loss. (**D**) *In vitro* growth curve of WT or the HA-deficient mutants in TSB medium (*n* = 6). (**E**) Quantification of capsular HA concentration (µg/mL), *n* = 5. (**F**) Survival curves of mice (*n* = 5) challenged with 1 × 10^9^ CFU of WT 15-Pm or the HA-deficient mutants. (**G**) Representative gross lung lesion. Images show the lungs from moribund WT-infected mice or mutant-infected mice that survived at 7 days post-infection via intratracheal instillation. PBS-inoculated mice served as the negative control. (**H**) Bacterial burdens across major organs and blood. CFU counts were log-transformed for analysis. For panels D and E, data are presented as mean ± SD, and statistical significance was determined using one-way ANOVA followed by Tukey’s multiple-comparison test. For panel H, data are presented as geometric mean ± geometric SD, and statistical significance was determined using two-way ANOVA followed by Šídák’s multiple comparisons test on log-transformed CFU/g or CFU/mL. Significance levels are indicated as follows: *P < 0.05, **P < 0.01, and ***P < 0.001.

To validate the feasibility of the pSET4s-mPheS plasmid for genetic manipulation, we targeted genes involved in capsular polysaccharide biosynthesis for deletion in swine-derived serogroup A *P. multocida*, given their well-defined functions, clear phenotypic associations, and established roles as key virulence determinants. In serogroup A strains, the capsule primarily consists of hyaluronic acid (HA), the biosynthesis of which is orchestrated by the *hyaBCDE* operon (3, 4). We utilized the pSET4s-mPheS plasmid to generate markerless deletion mutants of *hyaD* and *hyaE*, two key components of the *hyaBCDE* operon. Following the induction of plasmid and screening the double-crossover strains via p-Cl-Phe counterselection, putative mutants were identified by PCR (Fig. 3A and B). The deletion strains were further validated by Sanger sequencing, confirming the successful construction of the markerless Δ*hyaD* and Δ*hyaE* strains (Fig. S1A and B).

We next investigated the phenotype of capsule biosynthesis gene deleted mutants. On the TSA plate, both the Δ*hyaD* and Δ*hyaE* mutants exhibited rough and non-mucoid colony morphology compared to the WT 15-Pm (Fig. 3C), corroborating previous findings regarding their critical roles in capsule production (7). Remarkably, the *in situ* complemented strains (C∆*hyaD* and C∆*hyaE*) both fully restored the smooth and highly mucoid colony, same with the characteristic of the WT strain (Fig. 3C). In tryptone soya broth (TSB) medium, although the initial growth rates within 6 h remained comparable to the WT 15-Pm, both mutants exhibited a significantly reduced bacterial density during the stationary phase (Fig. 3D). Quantitative analysis also showed a substantial reduction in the HA concentration in the mutant strains, with the Δ*hyaD* mutant exhibiting a more pronounced decrease in capsule abundance compared to Δ*hyaE* (Fig. 3E). In agreement with the morphological restoration, the capsular HA content in both complemented strains successfully returned to wild-type levels (Fig. 3E), definitively attributing the capsule-loss phenotype to the targeted deletion of *hyaD* and *hyaE*.

The HA capsule of pathogenic bacteria is essential for evading host immune clearance and maintaining survival in the blood (8, 22). In murine models, HA deficiency markedly impaired bacterial virulence. Mice were challenged with 1 × 10^9^ CFU WT 15-Pm or HA-deficient mutant via intratracheal instillation. Mice challenged with WT 15-Pm were moribund within 60 h, while the Δ*hyaD* and Δ*hyaE* mutant-infected mice exhibited no clinical signs and survived more than 7 days post-infection (Fig. 3F). WT 15-Pm-infected mice showed severe pulmonary lesions characterized by increased lung volume with diffusely dark-red hemorrhagic areas (Fig. 3G). In contrast, the lungs of HA-deficient mutant-infected mice appeared normal, resembling the unchallenged control group (Fig. 3G). Bacterial load quantification of moribund mice further demonstrated that WT 15-Pm established colonization within the trachea and lungs. Subsequently, the pathogens tranverse the lung-blood barrier and cause systemic infection, leading to high bacterial burden in the liver, spleen, kidney, and blood (Fig. 3H). In contrast, the HA-deficient mutants were either cleared or with a lower burden at the initial infection site and liver (Fig. 3H). These results collectively demonstrate that the pSET4s-mPheS plasmid can serve as a reliable genetic tool for gene deletion in swine-derived serogroup A *P.multocida*, thereby providing a solid foundation for functional genomic studies in this important veterinary pathogen.

### Generation of a GFP-expressing *P. multocida* strain that retains virulence

**Fig 4:**
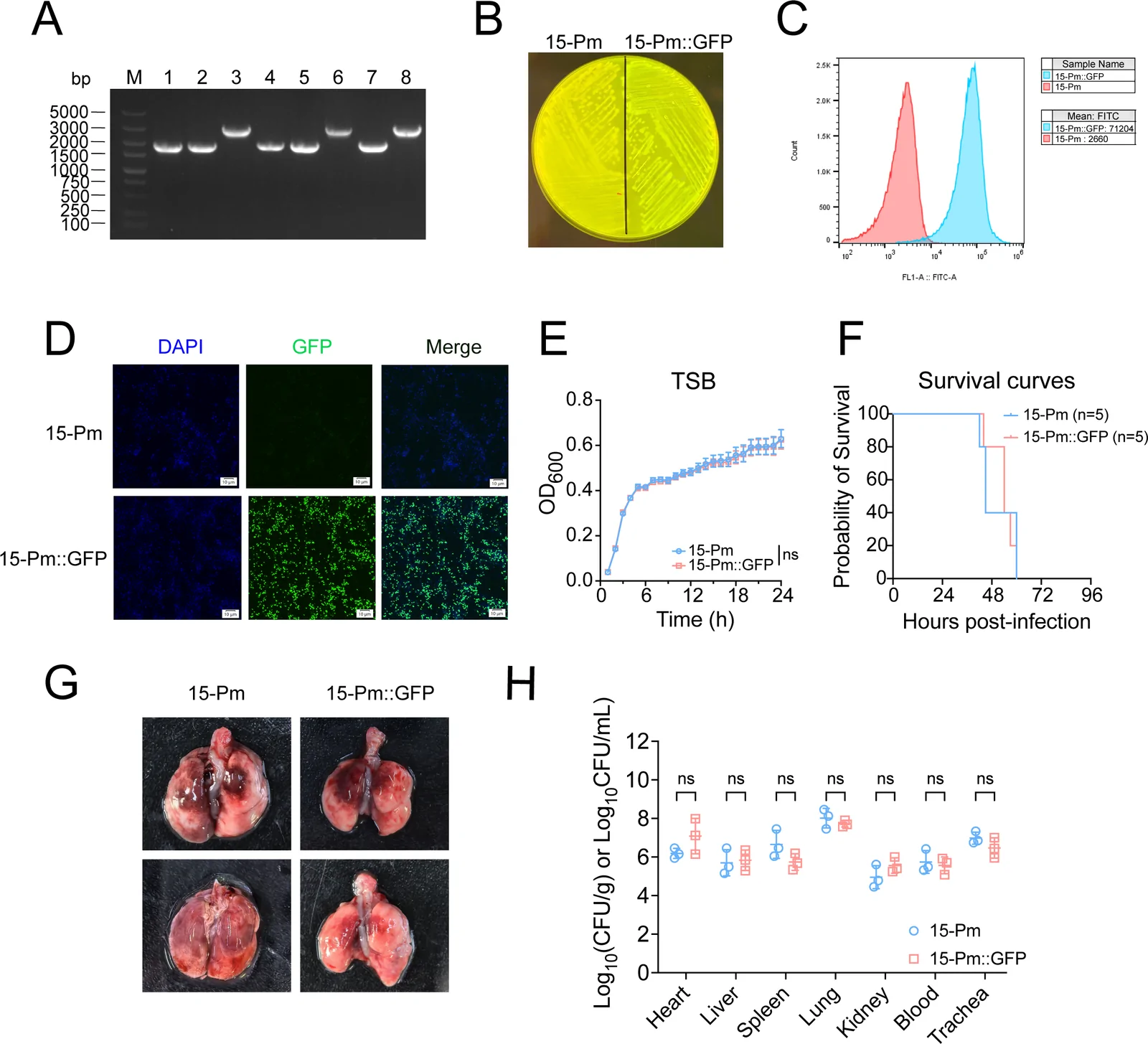
Construction and characterization of a GFP-labeled *P. multocida* reporter strain. (**A**) PCR-based verification of the 15-Pm::GFP mutants. Lane M: DL 5,000-bp DNA marker; Lanes 1–8: candidate GFP inserted clones; Lanes 1, 2, 4, 5, and 7: WT (1,610 bp); Lanes 3, 6, and 8: mutant (2,650 bp). (**B**) Macroscopic fluorescence phenotypes of WT 15-Pm and the 15-Pm::GFP reporter strain under blue light excitation (470 nm). (**C**) Flow cytometric analysis of fluorescence intensity. (**D**) High-resolution visualization of individual bacteria using fluorescence microscopy, confirming stable GFP signals at the single-cell level. (**E**) *In vitro* growth curve of WT or 15-Pm::GFP reporter strain in TSB medium (*n* = 6). (**F**) Survival curves of mice challenged with 1 × 10^9^ CFU of 15-Pm or 15-Pm::GFP (*n* = 5) via intratracheal instillation. (**G**) Gross lung lesions of moribund mice. (**H**) Bacterial burden in major organs and blood of moribund mice. For panel E, data are presented as mean ± SD. Statistical significance was assessed using two-way ANOVA followed by Tukey’s multiple-comparison test. For panel H, data are presented as geometric mean ± geometric SD. Statistical significance was assessed using two-way ANOVA followed by Šídák’s multiple comparisons test on log-transformed CFU/g or CFU/mL. Significance levels are indicated as follows: ns > 0.05.

To validate the knock-in efficiency of the pSET4s-mPheS plasmid in swine-derived *P. multocida* and to generate a fluorescent reporter strain for *in vivo* imaging, we integrated a *gfp* expression cassette into the 15-Pm genome between *nanM* and *dsbD_2* (Table S1). The *gfp* gene was placed under the control of the constitutive promoter P_manX_, a highly active promoter originally derived from streptococci (23). Following the same procedure as for gene knock-out, candidate strains were screened by PCR (Fig. 4A) and further confirmed by Sanger sequencing (Fig. S2C).

The GFP signal of the 15-Pm::GFP strain was robust across multiple platforms. On TSA plates, 15-Pm::GFP exhibited intense green fluorescence visible to the naked eye under blue light excitation (470 nm) (Fig. 4B). The fluorescence was further detected by flow cytometry and microscopy. Flow cytometric analysis showed that 15-Pm::GFP formed a distinct peak with a pronounced fluorescence shift compared to the WT 15-Pm, indicating high-intensity GFP expression (Fig. 4C). Consistently, strong fluorescence was observed under the FITC channel by fluorescence microscopy (Fig. 4D), highlighting the potential application of 15-Pm::GFP for in *vitro/vivo* imaging. These results collectively demonstrate that the pSET4s-mPheS plasmid can serve as a reliable genetic tool for gene knock-in in swine-derived serogroup A *P. multocida*.

To evaluate the potential impact of *gfp* gene integration on the pathogen’s biological and virulent characteristics, we compared the growth and virulence of 15-Pm::GFP with those of WT 15-Pm. The growth curves of the 15-Pm::GFP strain and the WT showed no significant differences in TSB medium (Fig. 4E). Mice infected with either the 15-Pm::GFP or WT strain exhibited 100% mortality within 60 hpi, with no significant differences in survival curves (Fig. 4F). Post-mortem examination showed that the 15-Pm::GFP strain induced pulmonary lesions comparable to those caused by the WT strain (Fig. 4G). Bacterial burdens in the lung, heart, liver, spleen, and kidney of 15-Pm::GFP-infected mice were equivalent to those observed in the WT-infected mice (Fig. 4H). These results suggest that the integration of the *gfp* gene and its constitutive expression do not significantly affect the *in vitro* growth, virulence, or organ colonization of *P. multocida*. The 15-Pm::GFP strain is, therefore, suitable for *in vitro* and *in vivo* tracking, largely reflecting the behavior of the WT strain and providing a useful tool for studying infection mechanisms of *P. multocida*.

### Organ sections imaging and *in vivo* tracking of 15-Pm::GFP in murine models

**Fig 5:**
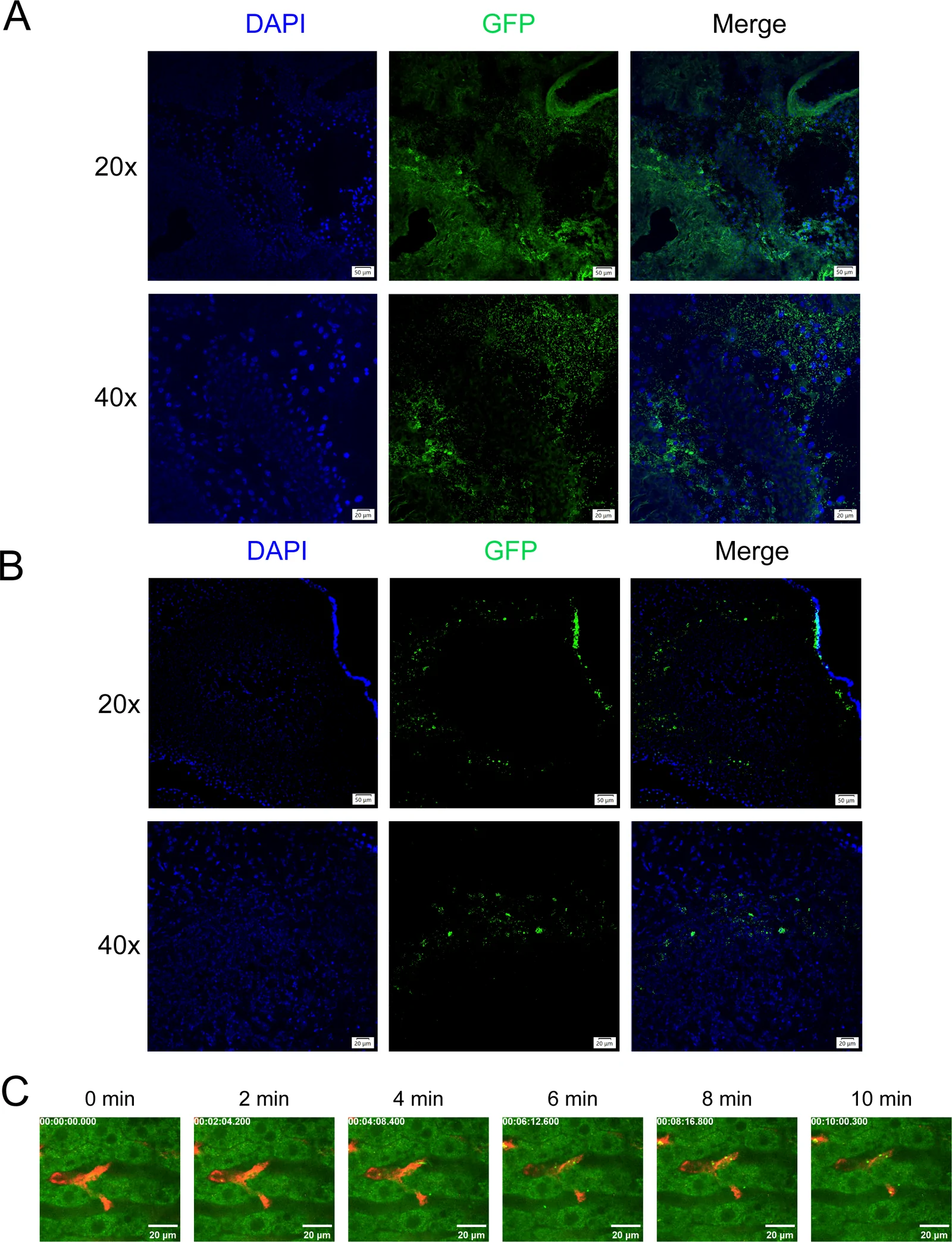
Spatiotemporal distribution and real-time *in vivo* dynamics of the 15-Pm::GFP infection. (**A**) Histopathological localization of 15-Pm::GFP within frozen sections of the lung, visualized by fluorescence microscopy. Nuclei are counterstained with DAPI (blue), and 15-Pm::GFP is shown in green, illustrating wide bacterial diffusion within the alveolar cavities. (**B**) Histopathological localization of 15-Pm::GFP within frozen sections of the liver. The imaging reveals characteristic bacterial clusters forming at the peripheral boundaries of hepatic necrotic foci. (**C**) Collection of frames from Movie S1 showing real-time *in vivo* imaging of *P. multocida* dynamics in the liver using spinning-disk confocal microscopy within 10 min post-intravenous infection (1 × 10^7^ CFU). Representative snapshots illustrate the rapid capture of 15-Pm::GFP (green) by resident Kupffer cells (red). All confocal and intravital images are representative of at least three independent biological replicates. Scale bars are indicated in each panel.

To evaluate the *in vivo* tracking performance of 15-Pm::GFP in infection experiments, as well as to assess the pulmonary distribution and hepatic infection dynamics of *Pasteurella multocida* in the mouse infection model, we performed high-resolution imaging of the 15-Pm::GFP reporter strain. Mice were challenged with 1 × 10^9^ CFU/mice via intratracheal instillation. Histopathological analysis of frozen sections from moribund mice revealed the precise anatomical localization of the pathogen within target organs. In the lungs, 15-Pm::GFP was observed to be widely diffused within the alveolar cavities (Fig. 5A).

In the liver, macroscopically visible multifocal necrotic lesions were identified on the surface. Fluorescence microscopy of these necrotic foci revealed that 15-Pm::GFP exhibited a distinct infection at the peripheral boundaries of the necrotic zones, forming bacterial clusters at the interface between healthy and lesion tissue (Fig. 5B).

To further investigate the real-time dynamics of infection, we utilized intravital spinning disk confocal microscopy to monitor the bacteria *in vivo*. Mice were challenged with 1 × 10^7^ CFU/mice via intravenous injection. Following injection, 15-Pm::GFP cells rapidly entered the systemic circulation (Movie S1). Notably, the fluorescent pathogens were actively captured by Kupffer cells in the liver within 10 min post-injection (Fig. 5C; Movie S1). These results collectively demonstrate that 15-Pm::GFP is a powerful tool for both *in vitro* tissue mapping and real-time *in situ* visualization of host-pathogen interactions, providing novel insights into dynamic and static *in vivo* tracking of *P. multocida*.

## DISCUSSION

We have established a robust markerless genetic manipulation plasmid for serogroup A swine-derived *P. multocida*, facilitating advanced functional genomic studies of clinical isolates. Current genetic tools for this pathogen are primarily reliant on *θ*-type replication, such as pACYCDuet-1, which exhibits a strict dependency on host-specific factors like RNase H and DNA Polymerase I (24). Such a dependency often results in a restricted host range and low plasmid stability in clinically isolated strains (e.g., pIG1 or pD70) (12, 25). Notably, our findings demonstrate that the RCR-based replication (pSET4s plasmid) exhibits superior compatibility across clinical serogroup A isolates. This advantage could be contributed by the RCR strategy, which bypasses the requirement for complex host-encoded initiation complexes by using its plasmid-encoded Rep protein and its cognate double-strand (*dso*) and single-strand (*sso*) origins (26). This mechanism is further supported by the characterization of the native *P. multocida* plasmid pOV, where autonomous replication is strictly driven by its self-encoded Rep protein (13). By utilizing this host-independent replication mechanism, we provided a toolkit for swine-derived isolates.

The initial transformation boundary observed between serogroups A and D prompted further investigation into the capsule. The clearing of the extracellular heparosan capsule via heparinase III successfully unlocked the delivery and functional replication of our RCR-type platform in strain serogroup D strain 11-Pm. In contrast, the *Enterobacteriaceae*-derived *θ*-type vector remained completely restricted even after capsule removal. We hypothesize this restriction resulted from native plasmid (9) or host defense mechanisms within serogroup D strain. Complete genome sequencing showed that strain 11-Pm carries a 5,279-bp endogenous plasmid, which is present in multiple *P. multocida* isolates. Further plasmid analysis via BLAST and MEME Suite indicated that this plasmid carrying a 38.5-kDa (978 bp) Rep protein features a replication origin with 22-bp direct repeats, which is the characteristic of a classic *θ*-type replicon (24, 27). In addition, a multilayered host defense network was identified in 11-Pm via the Defense-Finder pipeline (including Type III Restriction-Modification [R-M] and dual CRISPR-Cas platforms). These host systems routinely cleave the unmethylated foreign DNA produced in replication (28–30). Crucially, these dense defense systems do not stop our host-independent RCR machinery once the physical capsule barrier is removed. However, the resident native *θ*-plasmid likely causes severe resource competition or entry exclusion. Combined with active host restriction systems, these factors completely block enteric *θ*-type vectors. Similar to the conclusion of Ma et al*.* (31), our findings also demonstrate that the capsule is the primary physical barrier blocking DNA entry. Once this barrier is bypassed, our RCR-based architecture shows a clear replication advantage over standard enteric *θ*-replicons in overcoming host intracellular restrictions.

To enable high-resolution visualization of *P. multocida* infection dynamics, we developed a stable, chromosomally integrated fluorescence reporter strain. Unlike traditional plasmid-borne systems, which are often compromised by plasmid instability and the requirement for continuous antibiotic selection *in vivo* (21, 32), our approach utilized site-specific genomic integration. By inserting a *gfp* expression cassette between *nanM* and *dsbD_2*, we achieved high-intensity fluorescence in *P. multocida* without impairing its growth or systemic virulence. Beyond enabling simple localization, the GFP-expressing *P. multocida* strain allows direct visualization of the spatial distribution of infection. In contrast, conventional CFU quantification provides only a measure of bacterial burden while lacking critical information on the spatial organization of bacterial colonization. Furthermore, the real-time capture of 15-Pm::GFP by Kupffer cells within minutes post-infection provided a novel high-resolution insight into the early-stage systemic infection. Compared to whole-body bioluminescence, which offers macroscopic views but limited cellular detail, our high-brightness GFP *P. multocida* reporter strain facilitates single-cell resolution across opaque tissues. Collectively, this stable reporter strain provides *in situ* and intravital imaging data, enabling the tracking of both dynamic and static progression during *P. multocida* infections.

In summary, the genetic manipulation tool developed in this study successfully overcomes the challenges of engineering clinical swine-derived *P. multocida* isolates. The construction of a stable, chromosomally integrated fluorescence reporter strain provided a toolkit for real-time, high-resolution visualization of *P. multocida* infection kinetics. This study provides a useful tool for mechanistic dissection of key virulence determinants in *P. multocida*, enabling spatially resolved analysis of infection dynamics and supporting future studies on pathogenesis and intervention. In strict adherence to animal welfare ethics and the principles of minimizing animal use, *in vivo* systemic virulence evaluations were selectively restricted to the wild-type and capsule-deficient deletion strains, while redundant animal challenges for the complemented counterparts were intentionally omitted.

## MATERIALS AND METHODS

### Bacterial strains, plasmids, and culture conditions

The bacterial strains and plasmids used in this study are summarized in Tables S1 and S2. *Escherichia coli* DH5⍺ was employed for plasmid propagation and was cultured in Luria-Bertani (LB) broth at 37°C. *P. multocida* serogroups A clinical isolates were grown in tryptone soya broth (TSB, Oxoid, and CM0129B) at 28°C or 37°C as required.

Plasmids selected to evaluate replicon compatibility were as follows: RCR-type: pSET4s (Spc^r^), pDL278 (Spc^r^), and pSET2 (Spc^r^) and Enterobacteriaceae-derived *θ*-type (p15A/ColE1): pACYCDuet-1 (Cm^r^), pSHK5TS-TtAgoM2-HF (Amp^r^ and Kan^r^) and pUC19 (Amp^r^).

Antibiotics were added at the following concentrations for *E. coli and P. multocida* selection: spectinomycin (Spc 100 µg/mL), kanamycin (Kan, 50 µg/mL), ampicillin (Amp, 50 µg/mL), and chloramphenicol (Cm, 34 µg/mL).

### Mice

Six- to 8-week-old wild-type (WT) female C57BL/6 mice were housed in the Animal Experiment Center of Nanjing Agricultural University for 7 days prior to the experiment. Female mice were used to minimize variability and ensure consistency across all experimental conditions. The mice were randomly assigned to different experimental groups using random number generation, with each group including at least three biological replicates.

### Preparation of electrocompetent *P. multocida*

Electrocompetent cells of *P. multocida* were prepared using a modified glycerol-wash method (21). Briefly, an overnight culture was inoculated into TSB (1:100 dilution) and grown at 37°C with orbital shaking (180 rpm) until the optical density at 600 nm (OD_600_) reached 0.4–0.6. Hyaluronidase (10 mg/mL) was added to the culture at a 1:1,000 dilution, followed by a 30-min incubation on ice. The bacteria were harvested by centrifugation at 3,000 × *g* for 5 min at 4°C. The pellet was subsequently washed three times with ice-cold 15% (vol/vol) glycerol. Finally, the cells were resuspended in 15% glycerol to achieve a 100-fold concentration of the initial culture, aliquoted to 80 µL per tube and stored at −80°C.

For serogroup D isolates (such as strain 11-Pm), because the capsular matrix is predominantly composed of heparosan rather than hyaluronic acid, mid-logarithmic serogroup D bacterial cells were harvested by centrifugation, washed twice with sterile PBS, and resuspended in a specialized digestion reaction buffer (20 mM Tris-HCl [pH 7.0], 100 mM NaCl, and 1.5 mM CaCl_2_) prior to enzymatic digestion with heparinase III for 1 h. Following this targeted capsule-clearing incubation, the cells were immediately subjected to the identical downstream preparation parameters as described above, including three consecutive washes with ice-cold 15% (vol/vol) glycerol, 100-fold concentration resuspension, and storage at −80°C.

### Transformation evaluation assays

To ensure experimental consistency, all plasmids were purified using the E.Z.N.A. Plasmid Mini Kit I (Omega). For each transformation, 80 µL of the competent cells was mixed with 1 µg plasmid respectively in a pre-chilled 0.1-cm gap electroporation cuvette (Invitrogen). Electroporation was performed using a Gene Pulser X cell system (Bio-Rad) with the following optimized parameters: voltage = 2,500 V, capacitance = 25 µF, and resistance = 200 Ω. Following the pulse, 1 mL of TSB was added immediately to recover the competent cells. The cells were incubated at 28°C or 37°C as required for 3 h and then plated onto TSA plates supplemented with appropriate antibiotics. Transformation efficiency was calculated as the number of transformants per µg of plasmid (CFU/µg).

### Construction of pSET4s-based suicide plasmids

To construct the temperature-sensitive suicide plasmid pSET4s-sacB, the *sacB* cassette was amplified using primers P1/P2 with *Bacillus subtilis* genome as the template. The chloramphenicol promoter (P_cm_) was amplified by using primers P3/P4. The pSET4s-mPheS plasmid was constructed by amplifying the *mpheS* cassette using primers P5/P8 from pP_1503_-mPheS (17). The pSET4s vector was linearized by digestion with *Bgl II* (Takara). The target amplicons were cloned into the linearized vector by using the NEBuilder HiFi DNA Assembly Master Mix (NEB #M5520). All PCR products were purified using the E.Z.N.A. Gel Extraction Kit (Omega). The integrity of all constructed plasmids was verified by PCR and Sanger sequencing (Azenta). All primers used in this study are listed in Table S3.

### Counterselection sensitivity of sucrose and temperature sensitivity assays

To evaluate sucrose sensitivity, the wild-type *P. multocida* strain and its corresponding transformant harboring pSET4s-sacB were cultured overnight in TSB and then diluted 1:100 into fresh medium. Cultures were grown at 28°C with shaking (180 rpm) to OD_600_ at 1.0. Ten-fold serial dilutions were spotted onto TSA plates supplemented with 5%, 10%, or 15% (wt/vol) sucrose and incubated at 28°C. For p-chlorophenylalanine (p-Cl-Phe) sensitivity, the transformant strain harboring pSET4s-mpPheS and the wild-type strain were tested on TSA plates containing 10, 15, 20, or 25 mM p-Cl-Phe at 28°C. To evaluate the temperature sensitivity of the pSET4s, the transformant strain was grown to OD_600_ at 1.0, diluted, and spotted onto TSA plates containing 100 µg/mL spectinomycin. Plates were incubated at either 28°C (permissive temperature) or 37°C (non-permissive temperature) to assess plasmid clearance. All assays were performed in at least three independent biological replicates.

### Markerless gene deletion and insertion using mpheS

To generate Δ*hyaD* and Δ*hyaE* mutants, the upstream and downstream homologous flanking regions were amplified respectively and fused via Splicing by Overlap Extension PCR (SOE-PCR). The target amplicons were cloned into the *BamH* I/*EcoR* I sites of pSET4s-mPheS to yield pSET4s-mPheS01 (Δ*hyaD*) and pSET4s-mPheS02 (Δ*hyaE*). Similarly, for *gfp* insertion at the *nanM* locus, the *gfp* cassette was fused between *nanM* flanking arms and cloned to create pSET4s-mPheS03. For *in situ* genetic complementation of the deletion strains, fragments of the full-length wild-type hyaD or hyaE gene along with their upstream and downstream homologous flanking arms were amplified. To distinctively differentiate the complemented strains from the wild-type, specific synonymous point mutations (silent mutations) were engineered into the coding regions via mutagenic primers without altering the native amino acid translation. These altered amplicons were sequentially cloned into pSET4s-mPheS to yield the complementation vectors pSET4s-mPheS-ChyaD and pSET4s-mPheS-ChyaE.

The mutagenesis process followed a two-step allelic exchange strategy. (i) For first crossover (integration), the suicide plasmids were electroporated into the wild-type *P. multocida* strain. Transformants were selected on TSA plates with 100 µg/mL spectinomycin at 28°C. Single-crossover integration was promoted by subculturing transformants in TSB with spectinomycin at 37°C (non-permissive temperature) for two passages. Integration at the target locus was confirmed by PCR (primers P9/P14 and P12/P13 for *hyaD*; primers P15/P20 and P18/P19 for *hyaE*; P21/P26 and P24/P25 for *GFP*). (ii) For second crossover (excision), confirmed single-crossover strains were subcultured in antibiotic-free TSB at 28°C for several passages to encourage the second recombination event. The cultures were then plated onto TSA containing 20 mM p-Cl-Phe and incubated at 37°C for 24 h. Target colonies were screened by PCR (P9/P12 for Δ*hyaD*; P15/P18 for Δ*hyaE*; P21/P24 for 15-Pm::GFP) to identify double-crossover mutants. All mutations were further validated by Sanger sequencing.

### Determination of bacterial growth curves

Precultured 15-Pm and mutant strains were grown in TSB at 37°C to an OD_600_ of 1.0. The cultures were then inoculated into fresh TSB at a 1:100 dilution. The inoculated cultures were incubated at 37°C with orbital shaking at 180 rpm, and the OD_600_ was measured every hour using the BIOSCREEN C. All assays were performed in at least three independent biological replicates.

### Quantification of capsular hyaluronic acid

The concentration of capsular HA was determined according to previous research (33). The wild-type serotype A *P. multocida* strain and its mutants were cultured in TSB at 37°C to mid-exponential phase (OD_600_ = 0.4–0.6). Bacterial cells were harvested by centrifugation at 6,000 × *g* for 15 min. The pellets were suspended in PBS (pH = 7.4) at one-fifth of the initial culture volume. To gently strip the extracellular capsular polysaccharides without inducing cell lysis, the cell suspensions were maintained at 4°C and subjected to physical shearing within a homogenizer for 5 min (15 s on; 15 s off) without the addition of mechanical beads. The crude extracts were then centrifuged at 6,000 × *g* for 15 min at 4°C to collect the supernatant. A 50-µL aliquot of the supernatant was mixed with 50 µL of 0.1 M phosphate buffer (pH = 7.0) in a 96-well plate and incubated at 37°C for 15 min. Then, 100 µL of pre-warmed (37°C) CTM reagent was added to each well. After a further incubation at 37°C, the absorbance was measured at 600 nm using the Infinite 200 PRO microplate plate reader (TECAN). The HA concentration was calculated based on the standard curve generated with purified HA. All assays were performed at least three times with independent biological replicates.

### Flow cytometric analysis

To qualify the GFP expression in 15-Pm::GFP strain, bacterial cells were cultured to the mid-exponential phase (OD600 = 0.4–0.6), washed twice with phosphate-buffered saline (PBS, pH = 7.4). The bacteria were subsequently resuspended in PBS for analysis. The fluorescence intensity of the population was measured using a flow cytometer (Beckman Coulter, NJ, USA). A minimum of 10,000 events were captured for each sample, and data were analyzed using FlowJo software.

### Fluorescence microscopic observation of the 15-Pm::GFP with DAPI staining

15-Pm::GFP was grown in TSB at 37°C to an OD600 of 0.6–0.8. Hyaluronidase (10 mg/mL) was added to the culture at a 1:1,000 dilution, followed by centrifugation at 4,000 × *g* for 5 min. Bacterial cells were washed twice with PBS (pH = 7.4). A 10-µL aliquot of the bacterial suspension was spread on a glass slide, air-dried for 10 min, and fixed with 4% paraformaldehyde for 10 min at room temperature. The cells were stained with DAPI (1 µg/mL) in the dark for 10 min, rinsed with PBS, and blotted dry.

The coverslip was mounted on the glass slide using antifade mounting medium to minimize photobleaching. Fluorescence images were acquired using fluorescence microscopy (Zeiss, Axio observer) with excitation at 405 nm (blue, DAPI) and 488 nm (green, GFP). Three sets of images were captured for each sample: DAPI channel (bacterial nucleoid), GFP channel (GFP reporter in the strain), and a merged channel (overlay DAPI and GFP signals for colocalization analysis). Image processing were performed using ImageJ software, with consistent exposure time and gain settings applied across all samples to ensure comparability.

### Mouse challenge and determination of bacterial loads

The wild-type *P. multocida* strain and mutant strains were cultured in TSB at 37°C to an OD_600_ of 0.6–0.8. The bacterial cultures were harvested and washed three times with PBS (pH = 7.4) and resuspended to a final concentration of 2 × 10^10^ CFU/mL. Female C57BL/6J mice were challenged with 1 × 10^9^ CFU in 50 μL volume by intratracheal instillation. The clinical symptoms and survival time were monitored at predefined intervals. To minimize animal suffering, mice were euthanized with 10% sodium pentobarbital at time reaching ethically defined endpoints (moribund status), followed by the collection of major organs.

Organs were harvested, weighed, and transferred to homogenizer tubes containing 1 mL sterile PBS and homogenized thoroughly. Blood samples and tissue homogenates were serially 10-fold diluted with sterile PBS. A 10-μL aliquot of each dilution was spotted onto TSA plates and incubated at 37°C for 12 h. Colony-forming units (CFUs) were counted and normalized to tissue weight. Bacterial loads in tissues were expressed as CFU per gram (CFU/g), and bacterial loads in blood were expressed as CFU per mL (CFU/mL).

### Preparation and imaging of frozen organ sections

Organs harvested from moribund mice were immediately fixed with 4% paraformaldehyde for 24 h. The fixed tissues were dehydrated in 30% (wt/vol) sucrose at 4°C until they sank. The samples were then embedded in Optimal Cutting Temperature (OCT) compound and sectioned into 10-μm slices using a cryostat. The sections were then stained with DAPI and observed by fluorescence microscopy (Zeiss, Axio observer).

### *In vivo* imaging of hepatic bacterial dynamics

To observe real-time bacterial dynamics, 15-Pm::GFP was cultured in TSB as described above. The bacterial cultures were washed three times with PBS (pH = 7.4) and resuspended for the challenge assay. After anesthesia, the liver of mice was exposed and fixed for image. Kupffer cells were stained with PE-labeled anti-F4/80 antibody administered intravenously 30 min before challenge. Subsequently, 1 × 10^7^ CFU of 15-Pm::GFP was injected via the tail vein. The dynamic distribution of bacteria in the liver was observed by spinning-disk confocal fluorescence microscopy (Olympus SPINSR) with appropriate excitation wavelengths (488 nm for GFP; 560 nm for PE).

### Statistical analysis

All experimental assays were executed with at least three independent biological replicates, and animal challenge experiments were conducted using five or six mice per group, as specified in the corresponding figure legends. Given the multi-log span of microbial colonization and transformation yields, bacterial CFUs and transformation frequencies were log-transformed prior to statistical evaluation, with variance expressed as geometric mean with geometric standard deviation (geometric SD). For non-log-transformed data, results are presented as the arithmetic mean with standard error of the mean (SEM) or standard deviation (SD).

Statistical evaluations were performed using GraphPad Prism10 software. Comparisons across multiple experimental groups (e.g., tissue bacterial burdens and HA concentrations) were analyzed using one-way analysis of variance (ANOVA). Pairwise comparisons between two specific groups were assessed using an unpaired, two-tailed Student’s *t*-test. Statistical significance thresholds across all platforms were strictly defined as follows: *P < 0.05, **P < 0.01, ***P < 0.001$, and ns (no significant, *P* > 0.05).

## Data Availability

All data supporting the findings of this study are available within the paper. The data and materials from this study are available from the corresponding author upon reasonable request.

## SUPPLEMENTAL MATERIAL

Supplemental figures aem.00787-26-s0001.docx Fig. S1 and S2.

Supplemental tables aem.00787-26-s0002.docx Tables S1 to S3.

Movie S1 aem.00787-26-s0003.avi Real-time *in vivo* imaging of *P. multocida* dynamics in the liver using spinning-disk confocal microscopy within 14 min after intravenous infection (1 × 10^7^ CFU).

ASM does not own the copyrights to Supplemental Material that may be linked to, or accessed through, an article. The authors have granted ASM a non-exclusive, world-wide license to publish the Supplemental Material files. Please contact the corresponding author directly for reuse.
