# Supplementary material for: Development of a GFP-labeled Pasteurella multocida strain for in vivo organ infection tracing using a stable RCR-based genetic manipulation plasmid
Source: Appl Environ Microbiol. 2026 Aug 12;92(9):e00787-26. doi: 10.1128/aem.00787-26 (PMC13599540; doi:10.1128/aem.00787-26)
Supplement: Supplementary file 1 [file aem.00787-26-s0001.docx]

**Supplementary Figure S1: Structural architecture and PCR-based verification of the counter-selection suicide vectors.**

(A) Schematic maps of the suicide vectors pSET4s-sacB and pSET4s-mPheS. Plasmids were designed using SnapGene software. (B) PCR-based verification of the pSET4s-sacB by amplified the *sacB* cassette (1,682 bp). Lane M: DL2000 DNA marker; Lanes 1-5: candidate clones; Lane 6: negative control; Lane 7: positive control. (C) PCR-based verification of the pSET4s-mpheS by amplified the *mPheS* cassette (1,222 bp). Lane M: DL2000 DNA marker; Lanes 1-3: candidate clones; Lane 4: negative control; Lane 5: positive control.

**Supplementary Figure S2: Sanger sequencing validation of the capsule-deficient mutants, complemented strains, and fluorescence reporter strain.**

(A) Sanger sequencing chromatograms and alignment profiles confirming the 15-Pm Δ*hyaD* deletion mutant (top panel, highlighted in red) and its corresponding *in situ* complemented strain CΔ*hyaD* (bottom panel, highlighted in blue); (B) Sanger sequencing chromatograms and alignment profiles confirming the 15-Pm Δ*hyaE* deletion mutant (top panel, highlighted in red) and its corresponding *in situ* complemented strain CΔ*hyaE* (bottom panel, highlighted in blue); (C) Sanger sequencing validation of the genomic knock-in junction site in the 15-Pm::GFP fluorescence reporter strain.

**Supplementary Video．**Real-time *in vivo* imaging of *P. multocida* dynamics in the liver using spinning-disk confocal microscopy within 14 min post-intravenous infection (1×10^7^ CFU). Representative snapshots illustrate the rapid capture of 15-Pm::GFP (green) by resident Kupffer cells (red).
