# Supplementary material for: Development of a GFP-labeled Pasteurella multocida strain for in vivo organ infection tracing using a stable RCR-based genetic manipulation plasmid
Source: Appl Environ Microbiol. 2026 Aug 12;92(9):e00787-26. doi: 10.1128/aem.00787-26 (PMC13599540; doi:10.1128/aem.00787-26)
Supplement: Supplementary file 2 [file aem.00787-26-s0002.docx]

**Supplementary Table S1．Strains used in this study.**

| Strain | Relevant characteristic(s) | Source |
| --- | --- | --- |
| DH5α | For plasmid cloning | WeidiBio |
| 15-Pm | *P. Multocida*, Clinical isolate (serotype A ) | Laboratory collection |
| 1501-Pm | 15-Pm containing pSET4s-sacB plasmid, Spc^r^ | This study |
| 1502-Pm | 15-Pm containing pSET4s-mPheS plasmid, Spc^r^ | This study |
| 15-Pm Δ*hyaD* | *hyaD* deletion mutant in 15-Pm, constructed based on pSET4s-mPheS | This study |
| 15-Pm Δ*hyaE* | *hyaE* deletion mutant in 15-Pm, constructed based on pSET4s-mPheS | This study |
| 15-Pm::GFP | 15-Pm inserted a *GFP* gene downstream of nanM（A6J57_02910）, constructed based on pSET4s-mPheS | This study |

Spc^r^: Spectinomycin resistance

| Strain | Relevant characteristic(s) | Location | Source |
| --- | --- | --- | --- |
| 15-Pm | *P. Multocida*, Clinical isolate (serotype A ) | Anhui | Laboratory collection |
| 172-Pm | *P. Multocida*, Clinical isolate (serotype A ) | Yancheng，Jiangsu | Laboratory collection |
| 84-Pm | *P. Multocida*, Clinical isolate (serotype A ) | Shenqiu，Henan | Laboratory collection |
| 20-Pm | *P. Multocida*, Clinical isolate (serotype A ) | Dezhou，Shandong | Laboratory collection |
| 25-Pm | *P. Multocida*, Clinical isolate (serotype A ) | Dongying，Shandong | Laboratory collection |
| 155-Pm | *P. Multocida*, Clinical isolate (serotype A ) | Huanggang，Hubei | Laboratory collection |
| 7412-Pm | *P. Multocida*, Clinical isolate (serotype A ) | Anhui | Laboratory collection |
| 17-Pm | *P. Multocida*, Clinical isolate (serotype A ) | Fuyang，Anhui | Laboratory collection |
| 143-Pm | *P. Multocida*, Clinical isolate (serotype A ) | Ningbo，Zhejiang | Laboratory collection |
| 125-Pm | *P. Multocida*, Clinical isolate (serotype A ) | Huaian，Jiangsu | Laboratory collection |
| 32-Pm | *P. Multocida*, Clinical isolate (serotype A ) | Linyi，Shandong | Laboratory collection |
| 7413-Pm | *P. Multocida*, Clinical isolate (serotype D ) | Shandong | Laboratory collection |
| 123-Pm | *P. Multocida*, Clinical isolate (serotype D ) | Huaian，Jiangsu | Laboratory collection |
| 11-Pm | *P. Multocida*, Clinical isolate (serotype D ) | Anhui | Laboratory collection |
| 78-Pm | *P. Multocida*, Clinical isolate (serotype D ) | Dongying，Shandong | Laboratory collection |

**Supplementary Table S2. Plasmids uesd in this study.**

| Plasmid | Relevant characteristic(s) | Source |
| --- | --- | --- |
| pSET4s | RCR-type Plasmid selected to evaluate replicon compatibility, Spc^r^ | Lab stock |
| PDL278 | RCR-type Plasmid selected to evaluate replicon compatibility, Spc^r^ | Lab stock |
| pSET2 | RCR-type Plasmid selected to evaluate replicon compatibility, Spc^r^ | Lab stock |
| pACYCDuet-1 | *θ*-type Plasmid selected to evaluate replicon compatibility, Cm^r^ | Lab stock |
| pUC19 | *θ*-type Plasmid selected to evaluate replicon compatibility, Amp^r^ | Lab stock |
| pSHK5TS-TtAgoM2-HF | *θ*-type Plasmid selected to evaluate replicon compatibility, Amp^r^, Kan^r^ | Provided by Huazhong Agricultural University |
| pSET4s-sacB | pSET4s-based suicide vector containing sacB, Spc^r^ | This study |
| pSET4s-mPheS | pSET4s-based suicide vector containing mPheS, Spc^r^ | This study |
| pSET4s-mPheS01 | pSET4s-mPheS based suicide vector for deletion of *hyaD*, Spc^r^ | This study |
| pSET4s-mPheS02 | pSET4s-mPheS based suicide vector for deletion of *hyaE*, Spc^r^ | This study |
| pSET4s-mPheS03 | pSET4s-mPheS based suicide vector for insertion of *gfp* gene, Spc^r^ | This study |
| pSET4s-mPheS-ChyaD | pSET4s-mPheS based suicide vector for *in situ* complementation of *hyaD* gene in the ∆*hyaD* strain, Spc^r^ | This study |
| pSET4s-mPheS-ChyaE | pSET4s-mPheS based suicide vector for *in situ* complementation of *hyaE* gene in the ∆*hyaE* strain, Spc^r^ | This study |

Note: Spc^r^: Spectinomycin resistance; Cm^r^: Chloramphenicol resistance; Amp^r^: Ampicillin resistance; Kan^r^: Kanamycin resistance

**Supplementary Table S3．Primers used in this study.**

| Primers | Primer sequences (5′-3′) |
| --- | --- |
| P1 (*sacB*-F) | AATTTAGGAGGCATATCAAATGAACATCAAAAAGTTTGC |
| P2 (*sacB*-R) | GGTTTTCACCGTCATCACCGTTATTTGTTAACTGTTAATTGT |
| P3 (*Pcm*-F) | TGGTTTGATGTTGCGATTAATCATCACCGAACTAGAGCTTG |
| P4 (*Pcm*-R) | GCAAACTTTTTGATGTTCATTTGATATGCCTCCTAAATT |
| P5 (P-F) | AAATAAAAGCCCCCTGACGATGTTTCGCCAGAGGC |
| P6 (P-R) | GCTCGATGTTAGACATTATATTACTCTCCTTTGAGTTT |
| P7 (*mPheS*-F) | ACTCAAAGGAGAGTAATATAATGTCTAACATCGAGCAACA |
| P8 (*mPheS*-R) | AGCTGTGACCGTCTCCGGGATTAGAATTGTTCTGAGAAACGA |
| P9 (*hyaD* up-F for mut) | TAAAACGACGGCCAGTGAATTCAACCGCCATAGCCGAAACTT |
| P10 (*hyaD* up-R for mut) | TAATTTTTTAAGGACAGAAAAACACTTTGCATTTTATAAAA |
| P11 (*hyaD* down-F for mut) | TTAATAAAAATGCAAAGTGTTTTTCTGTCCTTAAAAAATTAA |
| P12 (*hyaD* down-R for mut) | CAGGTCGACTCTAGAGGATCCGTAAAACTCACTGCATCTGTTT |
| P13 (*hyaD*-F for single crossover) | CAATGGCTTCAATGAGATTTT |
| P14 (*hyaD*-R for single crossover) | TAGCACAATAAAAGAACGTC |
| P15 (*hyaE* up-F for mut) | AAAACGACGGCCAGTGAATTCTCTTCTAATAGCTCTGCAACATAAGA |
| P16 (*hyaE* up-R for mut) | TAAAGATTTTTTTAAGGACAGAAAATGAATACATTATCACAAGC |
| P17 (*hyaE* down-F for mut) | TCATTTTCTGTCCTTAAAAAAATCTTTATCTCTAATTTACA |
| P18 (*hyaE* down-R for mut) | CAGGTCGACTCTAGAGGATCCTTCTGTAAAGGTGTATCAATAGCG |
| P19 (*hyaE*-F for single crossover) | ATGATTCAAGCAAACTCGC |
| P20 (*hyaE*-R for single crossover) | GATTTCTGCTGTTGGAT |
| P21 (*GFP* up-F for mut) | AAAA CGACGGCCAGTGAATTCACACGTTCTCCTCGTTCAAC |
| P22 (*GFP* up-R for mut) | TGGATGAACTATACAAATAATGTTTATACAGCCCTTAAAT |
| P23 (*GFP* down-F for mut) | TTTCGGAAAAATCTTACGAAGCCCTTTTTTCGTTGCGTTA |
| P24 (*GFP* down-R for mut) | CAGGTCGACTCTAGAGGATCCGGAAACTGACATAATATGGA |
| P25 (*GFP*-F for mut) | ATTTAAGGGCTGTATAAACATTATTTGTATAGTTCATCCAT |
| P26 (*GFP*-R for mut) | TAACGCAACGAAAAAAGGGCTTCGTAAGATTTTTCCGAAAG |
| P27 (*nanM*-F for single crossover) | CTTTATGTGTTTGGTGTTT |
| P28 (*nanM*-R for single crossover) | AGTACGCCGCAGTATTGC |
| P29 (Pm-F) | ATCCGCTATTTACCCAGTGG |
| P30 (Pm-R) | GCTGTAAACGAACTCGCCAC |
| P31 (A-Pm-F) | TGCCAAAATCGCAGTCAG |
| P32 (A-Pm-R) | TTGCCATCATTGTCAGTG |
| P33 (D-Pm-F) | TTACAAAAGAAAGACTAGGAGCCC |
| P34 (D-Pm-R) | CATCTACCCACTCAACCATATCAG |
| P35 (C*hyaD*-up-F for complement) | AAAACGACGGCCAGTGAATTCAAAAAGTAAAACTCACTGCATCTG |
| P36 (C*hyaD*-up-R for complement) | AAATAATTTGAGTGCTAATTGATAGTCGTTGCTGTTATATGCT |
| P37 (C*hyaD*-down-F for complement) | GCATATAACAGCAACGACTATCAATTAGCACTCAAATTATT |
| P38 (C*hyaD*-down-R for complement) | GCAGGTCGACTCTAGAGGATCCGGGTAAACAATAACCGCCATA |
| P35 (C*hyaE*-up-F for complement) | AAAACGACGGCCAGTGAATTCTTCTGTAAAGGTGTATCAATAGCGT |
| P36 (C*hyaE*-up-R for complement) | TCTTGATAGTTAGACTGTTTATGTCCAATAATAATAACCTTTT |
| P37 (C*hyaE*-down-F for complement) | AAGGTTATTATTATTGGACATAAACAGTCTAACTATCAAGA |
| P38 (C*hyaE*-down-R for complement) | GCAGGTCGACTCTAGAGGATCCTCTTCTAATAGCTCTGCAACATAAG |

Note: Bases highlighted in red indicate the specific synonymous point mutations introduced into the primers.
